# Do public health nurses in Norway promote information on oral health?

**DOI:** 10.1186/1472-6831-11-23

**Published:** 2011-09-18

**Authors:** Marit S Skeie, Erik Skaret, Ivar Espelid, Nina Misvær

**Affiliations:** 1 Faculty of Medicine and Dentistry, University of Bergen, N-5020 Bergen, Norway; 2 Faculty of Dentistry, University of Oslo, Blindern, N-0327 Oslo, Norway; 3 Faculty of Health Sciences, Institute of Nursing, Oslo and Akershus University College of Applied Sciences, Norway

## Abstract

**Background:**

(i) to describe oral health counselling in Norway to parents with infants and toddlers, ii) to assess existing collaboration and routines in oral health matters between nurses and personnel in the PDS, iii) to evaluate to what extent oral health was integrated in the basic educational curriculum of public health nurses.

**Methods:**

This study was based on two separate surveys: the sample of Study I was 98 randomly selected child health clinics. A questionnaire covering oral health promotion counselling of parents with young children was returned by 259 nurses. Study II was a telephone survey addressing teachers of public health nurses at the eight educational institutions in Norway.

**Results:**

The response rate in Study I was 45%. Nutrition (breast feeding, diet) was the health subject most often prioritized in the counselling targeting parents of young children (by 60% of the nurses). Oral health was not among the first priority counselling subjects. The subject was seldom spontaneously mentioned by parents. Seventy percent of respondents reported (agreed or totally agreed) that they managed to provide information parents needed and 72% believed that the information they gave influenced parents' health behaviours. Seven nurses (5.2%) responded that they agreed with the statement that the information they gave only slightly influenced parents' health behaviour. Lack of time was mentioned as being a problem. Approximately half of the nurses (48%) had regular contact with the PDS for the 0-3 year-old children, but only a quarter of the nurses claimed that children's teeth were routinely examined at the child clinics. Some forms of previously established contact with the PDS enhanced the likelihood of nurses' referrals. Oral health was a minor part of the educational curriculum for public health nurses; at three institutions, the subject was totally absent.

**Conclusion:**

Collaboration between nurses and the PDS in Norway could be improved. Oral health should have a bigger place in the basic educational curriculum.

## Background

It is possible to control dental caries in young children (Early Childhood Caries, ECC) by adequate effective preventive strategies [1], but as teeth are more prone to caries shortly after eruption [2], the timing of preventive efforts is important. In young children with high caries activity, caries may develop even during tooth eruption. To succeed in prevention, it is thus essential to reach the preschool child and its caregivers during the eruption period of the primary teeth [3]. The first two years of a child's life have been suggested to be the most important period for effective interventions [4].

The Public Dental Service (PDS) in Norway normally recruits the children at the age of 3 years, but in 2003 it was reported that about one third of children attended for their first dental visit at PDS clinics at the age of four years [5]. This may indicate that the PDS does not prioritize early contact with toddlers to build rapport with parents. Effective caries prevention and early caries diagnosis in the primary dentition pre-supposes early contact before caries progresses.

According to the guidelines of the Directorate of Health [6], it is recommended that parents be informed about children's oral care and oral health promotion at the ages of 5, 7-8 and 11-12 months. In addition, the recommendation states that the child's teeth should be inspected at the age of two years, and the child referred to PDS if there are suspicions of caries, dental damage or other conditions needing specific information and counselling. The public health nurses working in child health clinics meet this group of young children and their caregivers regularly during their first two years. Nurses concerned about oral health promotion are a resource group for oral care, not only in the work of counselling but also in identifying children at high caries risk. They should therefore be seen as important collaborators for dental staff. During recent years, a growing interest in and a simultaneous recognition of nurses' role in oral health promotion have emerged [7].

To a great extent, parental behavioural factors are what determine the oral health status of their children. Studies providing evidence of the complexity of behavioural modifications after counselling have appeared [8]. In particular, it has been shown that to get the parents of at-risk infants to put into practice appropriate oral advice on a long term basis is a major challenge [9]. Even though a wide range of theoretical models of health behaviour exist [10-12], there is no unanimity about which is the most effective. This reflects how difficult it is, in counselling settings, to achieve individual behavioural change.

The aims of the present study were: i) to describe to what extent public health nurses in child health clinics provide information about oral health promotion to parents with 0-2-year-old children; ii) to assess the level of contact and exchange of oral health information currently practised between the public health nurses and the personnel in PDS. Specifically, we wanted to know whether the public health nurses examine children's teeth and how often children examined are referred to the PDS clinics; and iii) In addition, we wanted to assess how great a place oral health occupies in the basic educational curriculum for public health nurses.

## Methods

The present study was based on two separate surveys. Study I was based on a written questionnaire sent to Norwegian public health nurses who worked in child health clinics and were responsible for providing health care information to parents with 0 - 2-year-old children. When, in this report, the term "nurse" is used, it refers to public health nurses working in child health clinics. Study II was a telephone survey addressed to teachers at the eight institutions responsible for the education of public health nurses in Norway.

### Study I

#### Ethical approval

The study protocol was approved by the Regional Committee for Medical Research Ethics (REK). The protocol was evaluated by the Norwegian Social Science Data Services (NSD) which concluded that the study did not require their approval.

##### Sample selection

A sample of 98 child health clinics was randomly selected from the total number (826) in Norway. The list of health clinics was available from the Norwegian Directorate of Health, and the randomization managed by a sampling function created in Excel (Microsoft Corporation 2007). Altogether, 264 questionnaires were sent to the various child health clinics. Three letters with a total of five questionnaires were returned unopened.

##### Procedure

The 98 child health clinics were first contacted by phone and the survey was presented to the public health nurse in charge. She was informed about the purpose and the anonymity of the study and that it was supported by The Norwegian Directorate of Health. She was asked if she was willing to participate in the study by distributing questionnaires and prepaid return envelopes to the nurses working in her clinic. In addition to the questionnaires, the letters to the health clinics included a written note for the nurse in charge and another note for each of the nurses. A letter of appreciation and a reminder to possible non-responders were addressed to the nurse in charge two weeks after the first letter was sent.

##### Questionnaire

The structured self-administered questionnaire (Additional file 1) included questions about:1) Demographics; 2) Counselling routines and program for parents during the child's first and second year of age; 3) Oral health and nutrition; 4) Contact with PDS; and 5) Evaluation of own practices.

Demographic information included the nurse's year of birth, the number of years of experience as a public health nurse, and birth numbers in the area (included the proportion of children of non-western background). The counselling section included questions about the nurse's own choices of subjects to discuss and experiences about the topics parents wanted to discuss. Questions included were: 1) "How important do you think it is to inform parents about the following health subjects during the child's first year?" (child's development, sleep, vaccines, oral health, smoking, social interaction, nutrition, overweight, alcohol, child accidents, other subjects - suggestions?) rated on a 10-point scale (from not at all important to very important for each subject), and 2) "What health topics do the parents discuss spontaneously?" (child's development, sleep, vaccines, oral health, social interaction, nutrition, overweight, smoking, other subjects -suggestions?) ("mark the three most frequently selected subjects - from 1 to 3").

In the oral health and nutrition section, the nurses were told to report the most important oral health related subject and nutrition subject to include to parents with 1- and 2-year old children, respectively: "What is the most important oral health message you give to parents with 1 (2) -year-olds?" and "What is the most important advice about nutrition you give to parents with 1 (2)-year-olds?". They were asked to select one from a list of seven different options.

Three questions covered an evaluation of counselling program during the child's first year: 1) "I manage to give the type of health information the parents need", 2) "Health information given at the child health clinic influences the parents' behaviour only slightly", and 3) "I have sufficient knowledge about oral health to advise parents correctly" (quite agree, agree, neither agree nor disagree, disagree and completely disagree).

In the dialog with PDS section, the nurses were asked about types and regularity of the contact and to what extent they were referring children to the PDS. One last section included questions about a self-evaluation of the nurses' own oral health counselling, including sources of oral health related knowledge and routines about oral examinations of the children.

The frequency of referrals of children under three years (item 24 in the questionnaire) to the PDS, was transformed into a dichotomous dependent variable "Referrals" (Yes: often, sometimes. No: seldom, never) and used in bivariate and multiple logistic regression analyses. After exploring bivariate relationships between various items in the questionnaire and "Referrals", only significant predictors were allowed to enter the final multiple logistic regression model.

### Study II

This study was a standardized interview with one responsible person from each health educational institution for public health nurses in Norway (N = 8). The teachers in charge were asked about the educational program and to what extent oral health promotion was included. Those who were not reached by phone were contacted by e-mail. All the teachers were asked the following questions in the same sequence: 1) Is dental health a subject in the education program? (Yes/no); 2) If yes, how early in the educational program? (First terms/last terms); 3) If yes, how many hours? (Number of hours); and 4) Is any kind of oral health literature recommended? (Yes/no). All interviews were conducted during three weeks in October 2010.

### Statistical methods

All data management and analyses were performed using SPSS version 15.0 (SPSS Inc, Chicago IL). Chi-square statistics were employed to compare the groups. The level of statistical significance was set at 5 percent. Additionally, logistic regression statistics, bivariate and multiple forward stepwise analyses, were undertaken. The outcome measurements were odds ratios (OR) with 95% confidence intervals (CIs).

## Results

### Study I

One hundred and sixteen public health nurses completed and returned the questionnaire, constituting a response rate of 44.8% (116/259).

#### Demographic information

Table 1 presents the distribution of public health nurses according to age group and duration of public health nurse career. The mean age of the nurses was 47 years (range 28 to 64 years). The mean public health nurse career duration was 11 years (range 1 month to 35 years). The annual numbers of newborn in each area varied from 3 to 1028, with a mean of 60 children. Thirty per cent of the nurses (33/110) had 10% or more 1- year-old children of non-western immigrant origin. One nurse reported that all 1-year-olds in her region were of non-western immigrant background.

**Table 1:** Demographic information

Age distribution	
**Years**	**N (%)**

20-30	1 (0.9)
31-40	30 (25.8)
41-50	43 (37.1)
51-60	31 (26.7)
61 and more	11 (9.5)
*Total *	116 (100)

**Number of years working as a nurse**	

**Years**	**N (%)**

< 5	30 (26.1)
5-14	47 (40.9)
≥ 15	38 (33.0)
Total	115 (100)

#### Counselling routines to parents during the child's first year

During the child's first year of life, 69.4% (59/85) of the nurses provided health information according to the national recommendations [6] while 16.5% (14/85) only gave information in response to questions from the caregivers. Sixty per cent (59.5%) of the respondents (116) chose nutrition (breast feeding, diet) as first priority (Table 2), while 13.8% (15/116) chose development (physically and mentally) and 12.9% (16/116) communication abilities. Only one nurse responded that oral health was one of the most often chosen subjects.

**Table 2:** Counselling routines to parents during the child's first year

	N (%)
**Which health topics do you most often discuss?**	
Nutrition (breast feeding, diet)	69 (59.5)
Child development (physically and mentally	16 (13.7)
Communication	15 (12.9)
General health	5 (4.3)
Sleep	5 (4.3)
Oral health	3 (2.6)
Family situation and family health	2 (1.8)
Vaccines	1 (0.9)
*Total *	116 (100)

Sixty per cent (59.5%) of the respondents reported that nutrition (breast feeding, diet) was the subject the parents most frequently brought up for discussion. Three nurses responded that parents spontaneously chose oral health, and in one case it was prioritised third.

Among 10 given topics, the nurses chose oral health as the sixth most important subject (mean score 7.3), close to the subject smoking (7.1). Thirty-one nurses (26.7%) ranked oral health to be less important, and five ranked oral health to be not important at all.

#### Type of counselling program during the child's first year

Only four nurses (4/115) disagreed/totally disagreed with the statement "I manage to give the type of health information the parents need," while 70.4% reported that they did manage to give the information the parents needed (agree or totally agree) (Table 3). Neither age nor working experience were related to the answers given. More than sixty per cent (61.7%, 58/94) of the nurses gave lack of time as their response to the question "If you experience that you do not manage to give parents the health information they need, what are main reasons?", while 12.8% (12/94) wished that they had more knowledge. To the question "Health information given in the child health clinics influences the parents' behaviour only slightly", only 5.2% agreed and 0.9% quite agreed, while 22.4% of the nurses were indifferent to the statement.

**Table 3:** Type of counselling program during the child's first year

	N (%)
**Give your response to the following statement: **"**I manage to give the type of health information the parents need"**	
Totally agree	15 (13.0)
Agree	66 (57.4)
Neither agree nor disagree	30 (26.1)
Disagree	3 (2.6)
Totally disagree	1 (0.9)
*Total*	115 (100)

**Give your response to the following statement: "Health information given at the child health clinic influences the parents' behaviour only slightly"**	
Totally agree	1 (0.9)
Agree	6 (5.2)
Neither agree nor disagree	26 (22.4)
Disagree	67 (57.8)
Totally disagree	16 (13.8)
*Total *	116 (100)

#### Dental health and nutrition

The dental health information provided to parents of 1-and 2-yr-olds is presented in Table 4. "Between meals and at night, the child should have water only" was reported as the most important advice to parents of 1-year old children by 36.1% (39/108) of the nurses and to parents of 2-year-olds by 45.4% (49/108), followed by "avoid eating between meals", reported by 5.6% (6/108) and 14.8% (16/108) of the groups, respectively. When counselling about the subject of nutrition, sugar intake was rated as the most important topic for parents with 1- and 2- yr-olds by 3.6% (4/112) and 4.5% (5/111) of the nurses, respectively. The corresponding proportions of nurses choosing meal frequency as the primary nutrition message were 10.7% (12/112) and 5.4% (6/111).

**Table 4:** Oral health and nutrition

What is the most important oral health message you give to parents with 1-year-olds? (select one of the options)	
Avoid eating between meals	6 (5.6)
Between meals and at night, the child should have water only	39 (36.1)
Use of fluoride tablets (when the child can manage to suck them)	1 (0.9)
Avoid food with sugar	3 (2.8)
Start toothbrushing as soon as the first tooth erupts	58 (53.7)
I do not talk about oral health	0
Other subjects, in case what?	1 (0.9)
*Total*	108 (100)
**What is the most important oral health message you give to parents with 2-year-olds? (select one of the options)**	
Avoid eating between meals	16 (14.8)
Between meals and at night, the child should have water only	49 (45.4)
Use of fluoride tablets (when the child can manage to suck them)	10 (9.3)
Avoid food with sugar	16 (14.8)
Start toothbrushing as soon as the first tooth erupts	9 (8.3)
I do not talk about oral health	1 (0.9)
Other subjects, in case what?	7 (6.5)
*Total*	108 (100)

**What is the most important advice about nutrition you give to parents with 1-year-olds? (select one of the options)**	
Meal frequency	12 (10.7)
Diet composition	84 (75.0)
Sugar	4 (3.6)
Fat	0
Vegetable/fruit	1 (0.9)
Drink	8 (7.1)
Other topics, in case what?	3 (2.7)
I do not counsel about nutrition	0
*Total *	112 (100)

**What is the most important advice about nutrition you give to parents with 2-year-olds? (select one of the options)**	
Meal frequency	6 (5.4)
Diet composition	90 (81.1)
Sugar	5 (4.5)
Fat	0
Vegetable/fruit	2 (1.8)
Drink	3 (2.7)
Other topics, in case what?	4 (3.6)
I do not counsel about nutrition	1 (0.9)
*Total*	111 (100)

#### Contact with PDS

Regular contact with the PDS regarding 0 - 3 year-old children was confirmed by 49.1% (57/116) of the nurses (Table 5). Regular contact between the nurse and PDS was neither influenced by age nor by working experience. The mean number of contacts was one, while one nurse reported that she had been in contact with the PDS nine times. More than half of the nurses (54.8%, 63/115) never communicated with dentists or hygienists about children whose parents missed scheduled dental appointments in the PDS. Only 3.5% (4/115) often referred children to the PDS before three years of age, and 61.7% (71/115) did so occasionally. Nurses with longer experience (> 14 years) and with higher age (≥ 49 years) referred children to the PDS more often than those with less practice (p = 0.005) and lower age (p = 0.015). Nurses in charge of an area with a high proportion of immigrant children (above 20%) did not refer significantly more children than nurses with fewer immigrant children (p = 0.562).

**Table 5:** Contact with the Public Dental Service (PDS)

What type of contact do you have with oral health personnel in the PDS (children from 0 to 3 years)	
Regular contact	57 (49.1)
Only when I discover special problems	25 (21.6)
No contact	9 (7.7)
Other contact, (if so, what?)	25 (21.6)
*Total *	116 (100)
**Do you communicate with dentists and hygienists about children who miss scheduled dental appointments in the PDS**	
Often	1 (0.9)
Sometimes	30 (26.0)
Seldom	21 (18.3)
Never	63 (54.8)
*Total *	115 (100)

**Does it happen that you refer children to the PDS before the age of 3 years?**	
Often	4 (3.5)
Sometimes	71 (61.7)
Seldom	34 (29.6)
Never	6 (5.2)
*Total *	115 (100)

The following dichotomous variables showed significant relationship with the dependent variable "Referrals" in bivariate logistic regression analyses: "Communication about missed scheduled dental appointments in the PDS" (Yes: often, sometimes. No: seldom, never.) - OR 3.6 (CI: 1.3-10.3), "Contact with PDS more than once a year" (Yes/No) - OR 2.9 (CI: 1.1-8.4), "Working experience > 14 years" (Yes/No) - OR 2.6 (CI: 1.1-6.4). The persistent variable in the multiple model was "Communication about missed scheduled dental appointments in the PDS", showing an OR of 3.5 (CI: 1.2-9.9).

#### Evaluation of own practise

Self-assessment of "oral health knowledge" was based on response to the statement "I have sufficient knowledge about oral health to advise parents correctly" (Table 6). A total of 23.3% of the nurses were not sure if they had sufficient knowledge about oral health (neither agreed nor disagreed), and 3.4% disagreed with the statement. The results showed that the nurses who totally agreed (n = 24) were more experienced than the others. Compared with nurses with less working experience (< 15 years), there were significantly more nurses with long working experience (≥ 15 years) who "totally agreed" with the statement (9 vs. 15, p < 0.0001). The fact that they assessed themselves as having sufficient knowledge about oral health did not have any effect on routine control of children's teeth. The nurses who totally agreed (n = 24) with the postulate reported that they had received information from dentists/dental hygienists (n = 10), through the literature (n = 9) and from their education (n = 5). However, the dividing lines between the groups were not very distinct, as some nurses had gained information from more than one source.

**Table 6:** Evaluation of own practices

Give your response to the following statement: "I have sufficient knowledge about oral health to advise parents correctly"	
Totally agree	24 (20.7)
Agree	61 (52.6)
Neither agree nor disagree	27 (23.3)
Disagree	4 (3.4)
Totally disagree	0
*Total*	116 (100)
**Are the childs'teeth examined at the health centre?**	
Yes, always	28 (24.8)
Yes, sometimes	73 (64.6)
Seldom	9 (7.9)
Never	3 (2.7)
*Total*	113 (100)

One quarter of the nurses (24.8%, 28/113) said that children's teeth (1 - 2 yr) always were checked at the health clinic (Table 6). Sixty-seven per cent (66.7%, 56/84) reported that this examination was done by the nurses and 32.1%, (27/84) by the physicians. Regular contact with the PDS, age of the nurse or working experience, did not have any significant impact on the regularity of oral examination at the clinic. In the cases where the teeth were checked, it was less frequent at age 1 yr compared with age 2 yr (24.4% vs 72.2%). Caries was the principal disease for which the nurses searched (53.7%, 51/95). One nurse answered that she also was aware that neglect and abuse were associated with the oral cavity.

### Study II

Five institutions provided part-time education for public health nursing students during a period of two years (four semesters), while three had a full-time training course (two semesters) lasting one year. A total of 230 students graduated each year. The numbers varied from one institution to another from 30 to 60 students.

Oral health was included in the curriculum for public health nurse training in five of the eight institutions, either in the first term (1 institution), in the last term (3 institutions) or more occasionally (1 institution). The mean length of the program with lectures was three hours (range 2 - 4 hours). Five institutions confirmed that some dental health literature was recommended and four of those were institutions with an established education program including oral health.

## Discussion

This study was based on a randomized selection of public health nurses working in child health clinics in Norway. The aims were to describe oral health counselling to parents with infants and toddlers, to explore potential existing collaboration in oral health matters between nurses and personnel in the PDS regarding oral examinations of children and the frequency of referral of children with oral health problems to the PDS. An additional aim was to evaluate to what extent oral health was integrated in the basic public health educational curriculum in Norway. On a national basis, this study was the first randomized selected study focusing on the position of oral health in general health promotion provided by nurses in Norway.

The findings clearly showed that the nurses did not consider oral health to be among the first priority counselling subjects targeting parents of 0 - 2-yr-old children. Likewise, oral health was not a subject parents frequently talked about on their own initiative. Though the guidelines of the Directorate of Health [6] recommend nurses to include many different health topics in their counselling, seventy percent reported (agreed or totally agreed) that they managed to give the information the parents of 1-yr-old children needed. Further, most of the nurses (72%) believed that the information they gave, had an impact on parents' health behaviours. Seven nurses responded that they agreed with the statement that the information they gave, only to a small extent influenced the parents' health behaviour. Lack of time was mentioned as being a main barrier to successful counselling. Approximately half of the nurses (48%) had regular contact with PDS for the 0 - 3 year-old group of children. Contrary to the guidelines of Directorate of Health, children's teeth were not routinely examined at all child clinics; only a quarter of the nurses claimed this was done. The study also showed conclusively that oral health occupied a minor position in the educational curriculum for public health nurses; at several institutions, the subject was totally absent.

The major methodological limitation of this study was the low response rate and the lack of information about the non-respondents. The methodological strength was that the study was based on a randomized sampling, lowering some of the scientific concerns related to systematic differences between the respondents and the non-respondents. Nevertheless, this high proportion of non-responses in the study made it unwise to regard this study as a national survey, which was the original goal. Still some background information given from a group of The Norwegian Nurses Organisation's Professional Interest Group of Public Health Nurses tended towards representativeness. They could report that the mean age in the current Norwegian population of public health nurses (N = 2853) was 49 years, which corresponded to the mean age of the public health nurses participating in the study.

On the other hand, the low response rate may reflect the finding that nurses did not prioritize oral health.

The lack of theoretical models in the construction of the questionnaire has its natural explanations: a collaborative team with members from the Directorate of Health and the Faculty of Dentistry, University of Oslo, had requested that the focus be on the topics presented in the questionnaire. Future work aiming to reveal more in-depth knowledge about the nurses' role in oral health promotion will include questionnaires based on conceptual frames.

In spite of the limitations mentioned, the current data set provides important new information about the role of oral health in general health promotion for parents with 0 - 2 year-old children. The knowledge of what kind of oral health information nurses offer parents of infants and toddlers and routines they follow in oral health matters has hitherto been scarce.

Sixty per cent of the nurses in the present study chose nutrition (breastfeeding and diet) as the health subjects most frequently included in counselling during the child's first year, followed by child development. If the link between poor oral health, nutritional problems and being underweight [13,14] had been more frequently discussed and understated in media and in public, the nurses might have given oral health counselling higher priority. More emphasis of the fact that dental caries is a lifestyle disease [15], sharing risk factors with other diseases like obesity and diabetes, might well have enhanced interest in oral health. The document "Global goals for oral health 2020" [16] recommends the Common Risk Factor Approach in achieving the objectives of integrating oral health promotion and care in an overall strategy to influence health. A symposium on ECC in 2010 by the American Dental Association [17] has gone so far as to define ECC as a paediatric infectious disease with dental manifestations rather than a strictly dental disease. Such a definition might have led to a strengthening of the role of oral health promotion.

The majority of the nurses displayed self-confidence when giving information influencing parental behaviour. To achieve results in various settings, self-confidence is considered to be important. For example, with respect to parents' self-belief (parental efficacy), a study has demonstrated that parental beliefs control brushing and snacking habits, and actually were predictive of these behaviours [18]. According to some scientists, parental efficacy is believed to serve as a catalyst for initiating parental involvement [19]. It was Bandura [20] who, as early as 1989, introduced the original "self-efficacy" concept as "the subjective belief of the individual to be able to carry out a specific behaviour." On account of this, attention might be turned to enhancing the self-belief of those nurses who considered their own counselling as questionable in achieving behavioural change. It is nevertheless uncertain whether all those who believed they could influence parental behaviour succeeded, because the nurses participating in this study had not received any organized training in oral health promotion and counselling. It was also interesting to note that, concerning evaluation of their own practices, most of the nurses assessed themselves as having sufficient knowledge about oral health to advise parents correctly.

Advice about diet composition seemed to be the option most often selected in nutrition counselling with just a minor focus on sugar. This is worrying, because young children are known to be especially vulnerable to the destructive effects of sugar snacking [3,21] and caries activity is closely correlated with dietary practices [22]. On the other hand, it was a positive finding that so many nurses would recommend parents of toddlers to use water between meals and at night, and to start tooth-brushing as soon as the first tooth had erupted. The literature reveals that there is some confusion among nurses with regard to information about optimal fluoride exposure [23]. The responses in this study did not clarify whether the nurses were unsure or lacked confidence about fluoride policy. Competing demands for time during clinic visits has been mentioned in other studies as a common problem [7]. Other researchers have claimed that dental screenings make a good contribution to the overall oral health of young children and can easily be incorporated into a busy paediatric practice [24].

In disadvantaged groups with unfavourable parental oral attitudes, referrals to or collaboration with the PDS should include meeting mothers shortly after delivery, or even expectant mothers [25]. In Arkansas (USA), an ongoing free text message service provides weekly health tips for both pregnant women and new mothers. What is positive is that oral health is included among the topics, thereby signalling the fact that oral health is an integral part of general health[26].

When dental examinations were conducted, they mostly took place at the 2 - yr visit, which was in line with the published guidelines. Optimal preventive outcomes could be expected if the recommended initial dental inspection were advanced to 1 year of age. One might speculate that indifference to oral health in non-respondents could be one reason for not taking part in the study.

A US study focusing on the role of paediatric primary care providers in oral health promotion showed that as many as thirty per cent of children who had oral disease were not referred for dental care [24]. This may indicate communication obstacles between paediatric health workers and dentists. The present study was consistent with this finding. More than half of the nurses (52%) had no regular contact with the personnel in PDS about children 0 - 3 years of age, only four nurses said they *often *referred children to the PDS before 3 years of age, and almost three-quarters of the nurses reported that they seldom or never communicated with dentists or hygienists about children who missed scheduled appointments in the PDS. Additionally, the nurses with high proportion of children with immigrant background in their charge, populations with expected high caries burdens [27], did not refer a higher proportion to the PDS than other nurses. This is worrying, because in many minority groups, parental perceptions, oral health related knowledge and motivation for oral health are shown to be insufficient [28], underlining the need of those groups for support and guidance in oral health matters.

On a positive note, there are studies documenting that interventions in oral health training can have favourable direct impacts on ECC. A study performed at a paediatric outpatient clinic in USA has confirmed that a relatively brief intervention of counselling communication skill training was associated with increased provider knowledge and counselling. This again resulted in a significantly attenuated occurrence of ECC [7]. The authors also claimed that it was not enough to teach nurses oral health knowledge. If parents are to achieve a change in behaviour to reduce ECC risk, nurses must translate their acquired oral health knowledge into changed behaviour. To reduce disparities in treatment provided and outcomes between underprivileged groups and others, there must be greater focus on communication with parents, including cultural competency of care providers and the health literacy and health beliefs of parents. The barriers may be at the system level, the personal care level or the provider level [29].

Nurses' confidence to identify and to refer children appropriately with oral health problems has also been documented to be of importance [30], as well as knowledge about ECC and awareness of how important good oral health is for children's wellbeing and quality of life.

The information gathered in this study suggests that oral health is not seen as an integrated part of general health at all educational institutions. Three out of the eight national institutions did not have oral health in their educational curriculum. In light of this finding, it is understandable, however undesirable, that not all nurses are sufficiently engaged in oral health promotion to make it a priority subject in counselling. This finding was also in accordance with shortcomings regarding health schools in the UK, where policymakers at a national level have excluded oral health from guidance[31].

Approved guidelines in paediatric dentistry [32] are clear that early caries identification and interventions of non-invasive care are required to reduce the occurrence of ECC. Appropriate preventive measures may allow a natural arrest of caries while still confined to enamel [33]. To allow the identification of factors that identify individuals at highest risk of caries, the best choice is to see children prior to or very shortly after teeth have erupted. As oral health risk groups frequently include immigrants, these people form natural target groups for culturally tailored prevention strategies [34]. The current data set showed that established previous contact in one form or other with PDS enhanced the likelihood of nurses referring children before the age of three years. This is a reminder of the importance of collaboration between nurses and the personnel in the PDS.

## Conclusions

There is a need to strengthen the position of oral health in the basic educational curriculum of public health nurses and there is a need to enhance the collaboration between nurses and the PDS in Norway. Dentistry has a responsibility to support oral health promotion; dentistry should not take for granted that nurses automatically will provide caries risk assessment, ECC prevention for infants and toddlers and referrals to PDS. The PDS has also to manage its own problems, as noted elsewhere in dental health institutions [25]. It must be recognised that not all dentists consider it important to provide care for infants and toddlers, that many dentists are mostly emergency-oriented, and that some lack effective prevention strategies as working tools.

### Future research

In summary, nurses, together with paediatricians, represent one category of health personnel who can help address ECC by counselling to reduce risk. The style they adopt should include a supportive rather than judgemental approach if oral health behavioural change is to be achieved [8]. Giving instructions or advice alone does not modify attitudes or change health behaviours [35]. One promising patient-centred approach is motivational interviewing (MI) which has been shown to be a valuable instrument in improving both health and nutrition counselling [36], and also oral health [37]. A study among dieticians working in diabetes care, provided some evidence that brief training in MI was enough to induce changes in their counselling style [36]. Based on a review of models for individual oral health promotion and focusing on effectiveness, the conclusion was that MI was an approach with potential [8].

There is no guarantee that application of MI among nurses in counselling parents of infants and toddlers will prove itself to be effective; nevertheless, the strategy is promising and should be tried.

## Competing interests

The authors declare that they have no competing interests.

## Authors' contributions

All the authors contributed to the study idea, design, protocol and construction of the questionnaire. MSS: Entered the data, conducted the data analyses and contributed substantially to the manuscript writing. ES: Principle investigator, did the writing of the protocol and contributed substantially to data collection and manuscript writing. IE: Actively involved in the development of the study idea, design, data collection, literature review and writing of the manuscript. NM: Provided valuable comments to the paper in general and was actively involved with the design and procedure for the study. All the authors have read and approved the final manuscript.

## Pre-publication history

The pre-publication history for this paper can be accessed here:

http://www.biomedcentral.com/1472-6831/11/23/prepub

## Supplementary Material

Additional file 1 **The Questionnaire**. It is a structured self-administered questionnaire. Click here for file
