# Supplementary material for: Do public health nurses in Norway promote information on oral health?
Source: BMC Oral Health. 2011 Sep 18;11:23. doi: 10.1186/1472-6831-11-23 (PMC3189905; doi:10.1186/1472-6831-11-23)
Supplement: Additional file 1 — The Questionnaire. It is a structured self-administered questionnaire. [file 1472-6831-11-23-S1.DOC]

Public health nurses 2009 Reg.no.____________

**Questionnaire**

We hope you can find time to respond to the following questions:

**FIRSTLY, SOME BACKGROUND INFORMATION**

**1. Your year of birth________**

**2. How long have your worked as a nurse at a public health clinic?**

Number of years:______

**3. How many children are born per year in the area for which you are responsible (area of your responsibility)?**

Approximate number:______

**4. What proportion of 1-year-old children in your area are of non-western background (approximately)?**

Approx. proportion?:_________%

**ROUTINES CONCERNING HEALTH INFORMATION DURING THE CHILD’S FIRST YEAR OF LIFE**

| **5.** | **What routines do you have concerning choice of information on health topics during a child’s first year of life?**  Please tick one box | | | | | | | | |
| --- | --- | --- | --- | --- | --- | --- | --- | --- | --- |
|  | □ | Follow the topic list in the guidelines. | | | | | □ | Follow a list we have prepared at this public health clinic | |
|  | □ | Include the topics which I think should be transmitted? | | | | | □ | I inform about topics of concern to the parents | |
|  |  |  | | | | |  |  | |
| **6.** | **Which health topics do you most often discuss?**  (describe three to five topics in order of importance) | | | | | | | | |
|  |  | | | | | | | | |
|  |  | | | | | | | | |
|  |  | | | | | | | | |
|  |  | | | | | | | | |
| **7.** | **Which health topics do the parents discuss spontaneously?**  Mark three health topics which most often occur in order of importance (respectively 1, 2, and 3) | | | | | | | | |
|  |  | |  | | | |  |  | |
|  | □ | Child’s development | | | | | □ | Social interaction | |
|  | □ | Sleep | | | | | □ | Nutrition | |
|  | □ | Vaccines | | | | | □ | Overweight | |
|  | □ | Oral health | | | | | □ | Smoking | |
|  | □ | Other subjects, in case what | | | _________________________________________________ | | | | |
|  | □ | Parents seldom discuss on own initiative | | | | | | | |
|  |  |  | | | | |  | |  |
| **8.** | **On a scale from 1-10** (1 = not at all important, 10 = very important)  **- How important do you think it is to inform parents about the following health subjects during the child’s first year?**  Insert a number from 1 to 10 in front of each topic | | | | | | | | |
|  | □ | Child’s development | | | | | □ | Social interaction | |
|  | □ | Sleep | | | | | □ | Nutrition | |
|  | □ | Vaccines | | | | | □ | Overweight | |
|  | □ | Oral health | | | | | □ | Alcohol | |
|  | □ | Smoking | | | | | □ | Child accidents | |
|  | □ | Other subjects, in case what? | | | | __________________________________________ | | | |
|  |  |  | | | | |  |  | |
| **9.** | **How do you provide information for parents?**  Please tick one box which best suits your practise | | | | | | | | |
|  | □ | During conversation, I try to inform about all subjects | | | | | □ | I mainly inform about the subjects I feel are the most important | |
|  | □ | I usually base my information on the wishes and needs of the parents | | | | | | | |
|  | □ | Other things, specify | | __________________________________________________ | | | | | |
|  |  |  | | | | |  |  | |
|  |  |  | | | | |  |  | |

**ABOUT THE HEALTH COUNSELLING PROGRAM DURING THE CHILD’S FIRST YEAR**

| **10.** | **Please respond to the following statement: ”I manage to give the type of health information the parents need”**  Please, tick only one box | | | | | | | |
| --- | --- | --- | --- | --- | --- | --- | --- | --- |
|  | □ | | Totally agree | | | □ | Agree | |
|  | □ | | Neither agree nor disagree | | | □ | Disagree | |
|  | □ | | Totally disagree | | |  | | |
|  |  | |  | | |  | | |
|  |  | |  | | |  | | |
| **11.** | **If you experience that you do not manage to give parents the health information they need, what are the main reasons?**  Please, tick one box | | | | | | | |
|  | □ | | Lack of time for each parent | | | □ | The parents ask about things which I find irrelevant | |
|  | □ | | I wish I had more knowledge about some subjects | | | □ | The guidelines include too many topics | |
|  | □ | | Other causes, in case which? | | | ____________________________________ | | |
|  |  | |  | | |  | | |
|  |  | |  | | |  | | |
| **12.** | **Give your response to the following statement: “Health information given at the child health clinic influences the parents’ behaviour only slightly”**  Please, tick one box | | | | | | | |
|  | □ | | Totally agree | | | □ | Agree | |
|  | □ | | Neither agree nor disagree | | | □ | Disagree | |
|  | □ | | Totally disagree | | |  |  | |
|  |  | |  | | |  |  | |
|  |  | |  | | |  |  | |
| **13.** | **In the public health clinic at which you work, what is the typical age (years) of the child when a doctor present during the health counselling?** | | | | | | | |
|  | Please, write here::_________________________________________ | | | | | | | |
|  |  | | | | | | | |
|  |  | | | | | | | |
| **14a** | | **What is the most important oral health message you give**  **TO PARENTS WITH 1-YEAR-OLDS?**  Please, tick only one box | | | | | | |
|  | | □ | | Avoid eating between meals | | □ | Between meals and at night, the child should have water only | |
|  | | □ | | Use of fluoride tablets (when the child can manage to suck them) | | □ | Avoid food with sugar | |
|  | | □ | | Start toothbrushing as soon as the first tooth erupts | | □ | I do not talk about oral health | |
|  | | □ | | Other subjects, in case what? | | ______________________________________ | | |
|  | |  | |  | |  |  | |
|  | |  | |  | |  |  | |
| **14b** | | **What is the most important oral health message you give**  **TO PARENTS WITH 2-YEAR-OLDS?**  Please, tick only one box | | | | | | |
|  | | □ | | Avoid eating between meals | | □ | Between meals and at night the child should have water only | |
|  | | □ | | Use of fluoride tablets (when the child can manage to suck them) | | □ | Avoid food with sugar | |
|  | | □ | | Start toothbrushing as soon as the first tooth erupts | | □ | I do not talk about oral health | |
|  | | □ | | Other subjects, in case what? | |  |  | |
|  | |  | |  | |  |  | |
|  | |  | |  | |  |  | |
| **15a** | | **What is the most important advice about nutrition you give**  **TO PARENTS WITH 1-YEAR-OLDS?**  Please, tick only one box, but you may give more information on the same line (how often, how much, etc.) | | | | | | |
|  | | □ | | Meal frequency | | ______________________________________ | | |
|  | | □ | | Diet composition S | | ______________________________________ | | |
|  | | □ | | Sugar | | ______________________________________ | | |
|  | | □ | | Fat | | ______________________________________ | | |
|  | | □ | | Vegetables/fruit | | ______________________________________ | | |
|  | | □ | | Drink | | ______________________________________ | | |
|  | | □ | | Other subjects, in case what? | | ______________________________________ | | |
|  | | □ | | I do not talk about nutrition | | ______________________________________ | | |
|  | |  | |  | |  | | |
| **15b** | | **What is the most important advice about nutrition you give**  **TO PARENTS WITH 2-YEAR-OLDS?**  Please, tick only one box, but you may give more information on the same line (how often, how much, etc.) | | | | | | |
|  | | □ | Meal frequency | | | ______________________________________ | | |
|  | | □ | Diet composition S | | | ______________________________________ | | |
|  | | □ | Sugar | | | ______________________________________ | | |
|  | | □ | Fat | | | ______________________________________ | | |
|  | | □ | Vegetables/fruit | | | ______________________________________ | | |
|  | | □ | Drink | | | ______________________________________ | | |
|  | | □ | Other subjects, in case what? | | | ______________________________________ | | |
|  | | □ | I do not talk about nutrition | | | ______________________________________ | | |
| **CONTACT WITH THE PUBLIC DENTAL SERVICE (PDS)** | | | | | | | | |
|  | | | | | | | | |
| **16.** | **What type of contact do you have with oral health personnel in the Public Dental Service (PDS) (children from 0 to 3 years)?**  Please, tick only one box, | | | | | | | |
|  | □ | | Regular contact | | | □ | Only when I discover special problems | |
|  | □ | | No contact | | |  |  | |
|  | □ | | Other contact, (if so, what?) | | | ______________________________________ | | |
|  |  | |  | | |  |  | |
|  |  | |  | | |  |  | |
|  |  | |  | | |  |  | |
| **17.** | **If regular contact, how often**  **(number of times per year):** | | | | | | | |
|  |  | |  | | | ______________________________________ | | |
|  |  | |  | | |  |  | |
| **18.** | **Do you communicate with dentists and hygienists about children who miss scheduled dental appointments in the PDS**  Please, tick only one box | | | | | | | |
|  | □ | | Often | | | □ | Sometimes | |
|  | □ | | Seldom | | | □ | Never | |
|  |  | |  | | |  |  | |
|  |  | |  | | |  |  | |
|  |  | |  | | |  |  | |
| **EVALUATION OF OWN PRACTICES** | | | | | | | | |
| **19.** | **Give your response to the following statement: ”I have sufficient knowledge about oral health to advise parents correctly”**  Please, tick only one box | | | | | | | |
|  | □ | | Totally agree | | | □ | Agree | |
|  | □ | | Neither agree nor disagree | | | □ | Disagree | |
|  | □ | | Totally disagree | | |  |  | |
|  |  | |  | | |  |  | |
| **20.** | **If you agree or totally agree, where have you acquired the knowledge?** | | | | | | | |
|  |  | | Please, write here:_________________________________________ | | | | | |
| **21.** | **Are the child’s teeth examined at the health centre?**  Please, tick only one box | | | | | | | |
|  | □ | | Yes, always | | | □ | Yes, sometimes | |
|  | □ | | Seldom | | | □ | Never | |
|  |  | |  | | |  |  | |
| **22.** | **In case ”yes”, at which age is the dental examination usually performed?** | | | | | | | ______________________ |
|  | **- and if~~,~~ examined, by whom? (the public health nurse, the doctor, others?)** | | | | | | | ______________________ |
|  |  | | | |  |  | |  |
| **23.** | **In case ”yes”, what do you look for?**  Please, tick only one box | | | | | | | |
|  | □ | | Caries | | |  |  | |
|  | □ | | Other things, in case what? | | | ______________________________________ | | |
|  |  | |  | | |  |  | |
| **24.** | **Does it happen that you refer children to **the** PDS before the age of 3 years?**  Please, tick only one box | | | | | | | |
|  | □ | | Often | | | □ | Sometimes | |
|  | □ | | Seldom | | | □ | Never | |

***THANK YOU VERY MUCH FOR YOUR HELP!***
